# Evaluation of a short RNA within Prostate Cancer Gene 3 in the predictive role for future cancer using non-malignant prostate biopsies

**DOI:** 10.1371/journal.pone.0175070

**Published:** 2017-04-05

**Authors:** Karl H. Pang, Derek J. Rosario, Susan L. Morgan, James W. F. Catto

**Affiliations:** 1 Academic Urology Unit and Academic Unit of Molecular Oncology, Department of Oncology and Human Metabolism, University of Sheffield, Sheffield, United Kingdom; 2 Department of Histopathology, Royal Hallamshire Hospital, Sheffield, United Kingdom; University of Minnesota Hormel Institute, UNITED STATES

## Abstract

**Background:**

Prostate Cancer 3 (PCA3) is a long non-coding RNA (ncRNA) upregulated in prostate cancer (PCa). We recently identified a short ncRNA expressed from intron 1 of PCA3. Here we test the ability of this ncRNA to predict the presence of cancer in men with a biopsy without PCa.

**Methods:**

We selected men whose initial biopsy did not identify PCa and selected matched cohorts whose subsequent biopsies revealed PCa or benign tissue. We extracted RNA from the initial biopsy and measured PCA3-shRNA2, PCA3 and PSA (qRT-PCR).

**Results:**

We identified 116 men with and 94 men without an eventual diagnosis of PCa in 2–5 biopsies (mean 26 months), collected from 2002–2008. The cohorts were similar for age, PSA and surveillance period. We detected PSA and PCA3-shRNA2 RNA in all samples, and PCA3 RNA in 90% of biopsies. The expression of PCA3 and PCA3-shRNA2 were correlated (Pearson’s r = 0.37, p<0.01). There was upregulation of PCA3 (2.1-fold, t-test p = 0.02) and PCA3-shRNA2 (1.5-fold) in men with PCa on subsequent biopsy, although this was not significant for the latter RNA (p = 0.2). PCA3 was associated with the future detection of PCa (C-index 0.61, p = 0.01). This was not the case for PCA3-shRNA2 (C-index 0.55, p = 0.2).

**Conclusions:**

PCA3 and PCA3-shRNA2 expression are detectable in historic biopsies and their expression is correlated suggesting co-expression. PCA3 expression was upregulated in men with PCa diagnosed at a future date, the same did not hold for PCA3-shRNA2. Futures studies should explore expression in urine and look at a time course between biopsy and PCa detection.

## Introduction

Prostate cancer (PCa) is the most common male cancer in the western world [1]. Detection usually occurs through the identification of men at elevated risk, through serum PSA measurement, MRI or family history, and then prostate biopsy (PBx) [2]. Whilst biopsy is a well tolerated procedure usually conducted in the out patient setting, it carries considerable risks of low severity morbidity [3]. PSA is the commonest trigger initiating investigation for PCa, however the majority of men with an elevated PSA do not have PCa. In most series, around 20–30% of men with a raised PSA have PCa on initial biopsy [2]. Patients without cancer in their initial biopsy and persistently elevated PSA levels are difficult to manage. Whilst 10–30% have undetected PCa [4, 5], automatic repeat PBx (rptPBx) represents over-investigation for many and carries the risks of additional procedures.

There are few agreed protocols for deciding upon identification of men needing rptBx to diagnose PCa or with active surveillance regimens [6] [7]. Whilst MRI now appears a promising tool in this context, there is an urgent need to identify biomarkers that may also inform this decision. With this role in mind, Prostate Cancer 3 (PCA3) was introduced into clinical practice. PCA3, is a long non-protein coding RNA (ncRNA) that is over-expressed in >95% of PCa [8]. PCA3 is located on chromosome 9q21-22, within the BMCC1/PRUNE2 gene, in an antisense orientation [9, 10]. PCA3 mRNA expression can be detected in urine samples from men with PCa and has a higher specificity than PSA for cancer detection (PROGENSA assay, [11]). As such, the PCA3 score (PCA3/PSA mRNA ratio x 1000) is higher in men with PCa and can be used to stratify the use of rptBx [12, 13].

PCA3 appears a promising biomarker, its widespread application is limited by analytical concerns and a lack of knowledge about its function [14]. With regards to the former, the assay detects a 380 base RNA transcript that is unstable *ex vivo*. Biological measurement requires sample protection from RNA degradation and transport to certified analytical laboratories, making the test expensive and vulnerable to transportation errors.

We recently identified a short hairpin ncRNA, termed PCA3-shRNA2, expressed from intron 1 of PCA3, whose expression was closely correlated to PCA3 [15]. PCA3-shRNA2 is stable *ex vivo* and so may overcome some of the risks of the PROGENSA assay. Furthermore, we identified regulatory targeting by this ncRNA, of mRNAs involved in PCa biology (including COPS2 and SOX11), suggesting one oncogenic function for the PCA3 locus. It is known that many short ncRNAs act through the regulation on larger mRNAs [16].

Our initial report used tissue and urine samples from men with and without PCa. All specimens were taken at first presentation or diagnosis. However, the PROGENSA assay is clinically advocated for guiding rptBx in men with an elevated PSA. Here we test the predictive role of PCA3-shRNA2 in men with and without PCa, whose initial biopsy did not detect cancer.

## Materials and methods

### Patients and samples

The Royal Hallamshire Hospital is the sole urological provider for the city of Sheffield, UK. We searched the pathology database for men with an initial PBx between 1994 and 2010 (to allow follow up). We annotated with clinical details, the number and timing of rptBx, and the eventual diagnosis of PCa. We identified men whose initial PBx did not show cancer, and selected a matched cohort whose rptBx did or did not find PCa. This study was approved by the local Ethics Committee (Trent Multicentre Research Ethics Committee HTA 96/20/99).

### RNA extraction from prostate biopsies

We obtained the formalin fixed paraffin embedded (FFPE) blocks from the initial PBx and cut sections at 10um thickness. We stained one section with H&E to confirm diagnosis and extracted RNA from the remaining. We removed paraffin (deparaffinization solution, Qiagen, UK) before lysis with Proteinase K. Tissue microdissection was not used. Samples were treated with DNase to eliminate all genomic DNA, before washing and elution in RNase-free water. Total and miRNA were extracted using miRNeasy FFPE kit (Qiagen, UK) as per manufacture’s protocol and measured using a 2100 Bioanalyzer (Agilent).

### Measurement of RNA expression

As detailed [15], extracted RNA was subject to real-time quantitative RT-PCR (HT7900 PCR system) using the high-capacity kit Reverse Transcription cDNA kit (Applied Biosystems) and the TaqMan microRNA Reverse Transcription kit (Applied Biosystems). RNA expression was determined using qPCR with TaqMan primers for PSA (Assay ID: Hs03063374_m1), PCA3 (Assay ID: Hs03309852_g1) and two custom designed TaqMan assays, PCA3-shRNA2A (ACTGCACTCCAGCCTGGGCA (Ambion: assay IDs, CSGJ090)) and PCA3-shRNA2B (CACTGCACTCCAGCCTGGGCA (Ambion: assay IDs, CSHSNF8)). Expression of PCA3, and PCA3-shRNA2 was normalized to PSA calculated using DCt values [17].

### Power calculation and statistical analysis

We estimated that 10–15% of men with rptBx have PCa [4, 5] and that PCA3 increases this detection rate to 25% [18]. With a power (1-β) of 0.8 and a significance of 0.05, we calculated around 200 men were needed in our study. Following analysis, RNA expression in prostate tissues was compared between men with and without cancer using Student’s t-test or ANOVA, and correlated between species using Pearson’s coefficient. The ability of each RNA to detect PCa was calculated using concordance indices and plotted using ROC curves [19]. All analyses were two sided and conducted within SPSS Vsn. 23.0 (SPSS Inc, Illinois). Graphs were plotted using PRISM 6.0 (GraphPad Software inc.). A p value of <0.05 taken as the threshold of significance.

## Results

### Patients and samples

We obtained residual tissue from the first PBx (between 2002 and 2008) of 116 men with an eventual diagnosis of PCa and 94 men without PCa (Table 1). The two populations were broadly comparable for clinical features. The mean (± st. dev.) age at referral within our cohort was 63.5 (±7.1) for men with cancer and 62.5 (±6.5) years for those with benign PBx. The mean initial PSA was 9.5 (±1.8) and 13.2 (±61.4) in the cancer and benign group respectively. A total of 17/23 (73.4%) men with suspicious findings and 4/7 (57.1%) men with HGPIN on initial PBx were found to have cancer on rptBx. The majority of cancers (n = 74, 64%) were detected on the second PBx and Gleason score 3+3 = 6 was the most common grade amongst these tumors (n = 78, 67.2%). The mean (SD) time to diagnosis of PCa from the initial negative PBx was 29.8 (±36.6) months.

**Table 1:** Patients and samples analyzed in this report.

		Eventual diagnosis				
		Cancer		Benign		
		n	%	n	%	p-value
Total		116	55.2	94	44.8	
Age at referral: Mean (± st.dev)		63.5 ± 7.1		62.5 ± 6.5		0.25
Initial PSA: Mean (± st. dev)		9.5 ± 1.8		13.2 ± 61.4		0.56
Referral to diagnostic biopsy (months): Mean (± st. dev)		29.8 ± 36.6		25.1 ± 29.6		0.97
Initial biopsy						
	Benign prostate	95	81.9%	84	72.4%	
	ASAP	0	0.0%	1	0.9%	
	PIN	4	3.4%	3	2.6%	
	Suspicious	17	14.7%	6	5.2%	0.16
No. biopsies						
	2	74	63.8%	65	56.0%	
	3	25	21.6%	23	19.8%	
	4	14	12.1%	6	5.2%	
	5	3	2.6%	0	0.0%	0.20
Gleason Score						
	3+3 = 6	78	67.2%	NA		
	3+4 = 7	17	14.7%	NA		
	4+3 = 7	10	8.6%	NA		
	8–10	11	9.5%	NA		NA

### RNA expression in prostate biopsies

**Fig 1:**
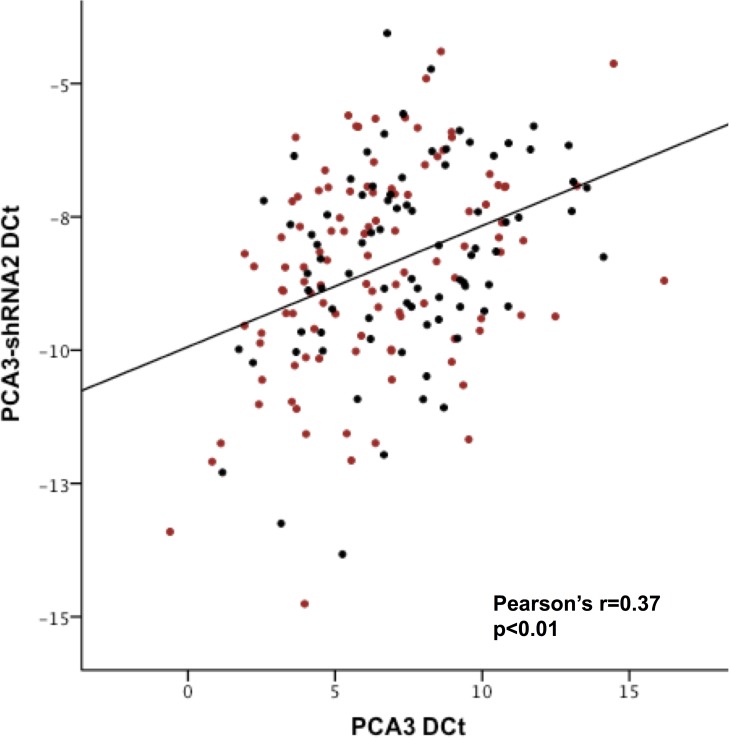
Scatterplot of PCA3 and PCA3-shRNA2 RNA expression normalized to PSA mRNA in formalin fixed paraffin embedded benign prostate biopsies. *Red, cancer detected on repeat biopsies (black, negative repeat biopsies)

We detected PSA and PCA3-shRNA2 RNA in all samples, and PCA3 mRNA in 190 (90%) biopsies. Expression of PCA3 and PCA3-shRNA2 was normalized to PSA mRNA, as for the PROGENSA assay. As seen previously, expression of PCA3 and PCA3-shRNA2 were correlated (Pearson’s r = 0.37, p<0.01, Fig 1) suggesting co-expression. Quantification relative to PSA mRNA showed higher expression of the shRNA than PCA3 (average ± st. dev = 373,829 ±228,546 fold), suggesting stability of the shorter RNA and easier detection. Interestingly, there appeared no deterioration in RNA yield across the time period that the samples were stored (S1 Fig).

### RNA expression and eventual diagnosis

**Fig 2:**
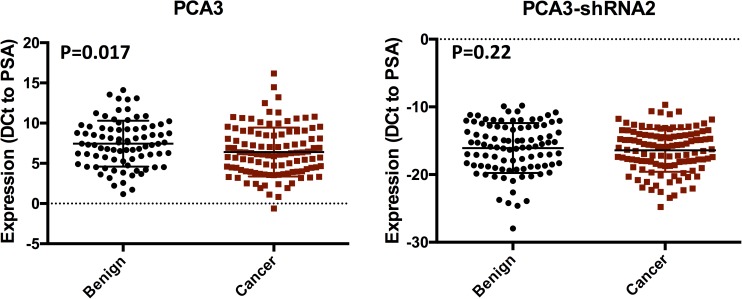
Bot plot of PCA3 and PCA3-shRNA2 expression (shown as DCt normalized to PSA mRNA) stratified for eventual diagnosis. Lines represent Mean and Standard Deviation. *p-value = Student’s t-test.

**Fig 3:**
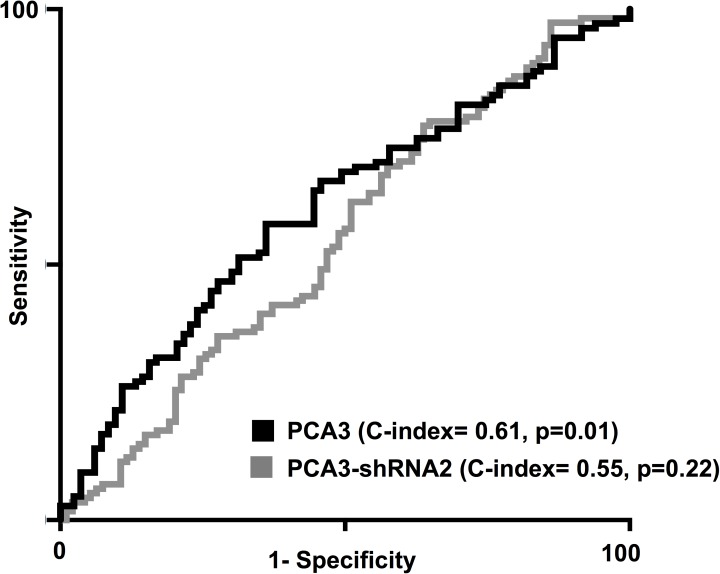
Predictive ability of PCA3 and PCA3-shRNA2 expression for the eventual diagnosis of prostate cancer.

We compared RNA expression with the eventual diagnosis in each man (Table 2 and S2 and S3 Figs). We saw upregulation of PCA3 (average 2.1 fold) and PCA3-shRNA2 (average 1.5 fold) in men with an eventual diagnosis of cancer, when compared to those with only benign histology (Table 2). For PCA3, this difference reached statistical difference (t-test p = 0.02), but this was not the case for PCA3-shRNA2 (p = 0.2, Fig 2). The expression of PCA3 and PCA3-shRNA2 did not vary significant when stratified by time of prostate cancer diagnosis from initial biopsy (S2 Fig). To determine predictive role, we calculated the concordance index for each RNA (Fig 3). Whilst, PCA3 was associated with the detection of PCa (C-index 0.61, p = 0.01), this was not the case for PCA3-shRNA2 (C-index 0.55, p = 0.22).

**Table 2:** RNA expression stratified by eventual diagnosis.

	Eventual diagnosis					
	Cancer		Benign		Fold change**	t-test
	Mean DCt*	± St. Dev	Mean DCt*	± St. Dev		p-value
PCA3-shRNA2	-16.53	3.24	-15.99	3.61	1.45	0.20
PCA3	6.40	3.03	7.48	2.86	2.12	0.02

*DCt normalized to PSA mRNA

**Fold change in patients with cancer vs. those with BPH

## Discussion

We previously identified a novel short ncRNA expressed within the host PCA3 and BMCC1 loci [15]. In fresh frozen prostate tissues and in urine samples obtained following prostatic massage, we identified that the expression of PCA3 and shRNA2 were closely correlated, and both were associated with the presence of PCa. Expression was not restricted to prostatic cell lines and was present at lower levels in men without PCa. However, the clinical context of the PCA3 assay is to guide further investigation in men with an elevated serum PSA, whose PBx do not reveal cancer. With this in mind, we undertook the current study. Whilst the PCA3 assay uses urine samples to measure PCA3, this resource was not available, and others have found that urine PCA3 expression reflects that within tissues [11].

We selected men with and without PCa, matched for age, PSA and duration of rptBx, and extracted RNA from stored FFPE tissue not used during pathological reporting. We identified several key findings. Firstly, despite storage at room temperature for 8–14 years, we detected robust expression of PSA and PCA3 mRNA, and the short ncRNA (PCA3-shRNA2). The latter was most abundant, in keeping with its stability and ease of detection. Secondly, we identified correlated expression of the two RNAs expressed from the PCA3 gene confirming our previous observations and suggesting that the short ncRNA is a product of PCA3 transcription [15]. These two observations suggest that PCA3-shRNA2 may be incorporated into the PCA3 assay to facilitate easier handling of the biological samples prior to laboratory measurement.

However, our experiments failed to support our primary hypothesis, namely that PCA3-shRNA2 expression was associated with a subsequent diagnosis of PCa. This is in contrast to expression of PCA3 mRNA within our population, and may reflect that this ncRNA is not expressed by PCa, that ncRNAs have a dynamic expression that is less stable than mRNAs over time, or that our experimental design was wrong. With regards to the former, we previously found high malignant expression of PCA3-shRNA2 in 3 separate cohorts (i.e. PCa cell lines, fresh frozen microdissected prostatic tissues and prostate massage fluids) suggesting this may not be the explanation. With regards to dynamic expression, it is known that one function of short ncRNAs is to epigenetically regulate mRNA expression. This regulation is dynamic, with ncRNA expression fluctuating depending upon the cellular needs and stress. For example, individual ncRNAs have been found to have both oncogenic and tumor suppressor roles, depending upon the context [20]. Thus, it is plausible that 2–3 years before the diagnosis of cancer, PCA3-shRNA2 expression is not elevated, as the target mRNAs (such as COPS2 and SOX11) do not have, as yet, altered function in the prostate. With regards to experimental design, we used whole biopsy tissues without micro or macro-dissection which may have influenced the quality of our samples. We powered the study using expression estimates and used FFPE tissues (to replace urine samples). We did find a trend towards upregulation of PCA3-shRNA2 in cancer, suggesting underpowering of the sample size. It may also be that FFPE tissues do not preserve differential expression of all RNAs. Of note, previous analyses of PCA3 expression in PBx tissues have reported inconsistencies, with both upregulation in cancer and no difference between malignant and normal prostate [21, 22] [23].

## Conclusions

Here we have found stable expression of PSA, PCA3 and PCA3-shRNA2 in historic prostate biopsy samples. Whilst PCA3 and PCA3-shRNA2 expression were correlated, only the former was significantly associated with the presence of occult PCa. To clarify these conflicting results, futures studies should have a larger sample size and analyze expression with respect to time before the diagnosis of cancer.

## Supporting information

S1 Fig PSA, PCA3 and PCA3-shRNA2 expression in formalin fixed paraffin embedded prostate biopsies stratified by year of collection. (PDF)

S2 Fig PSA, PCA3 and PCA3-shRNA2 expression in formalin fixed paraffin embedded prostate biopsies stratified by time to diagnosis of prostate cancer from initial prostate biopsy (PDF)

S3 Fig Box plot of PCA3 and PCA3-shRNA2 expression (DCt normalized to PSA mRNA) stratified for Gleason Scores. (PDF)
