# Supplementary figures and images for: Evaluation of a short RNA within Prostate Cancer Gene 3 in the predictive role for future cancer using non-malignant prostate biopsies
Source: PLoS One. 2017 Apr 5;12(4):e0175070. doi: 10.1371/journal.pone.0175070 (PMC5381913; doi:10.1371/journal.pone.0175070)

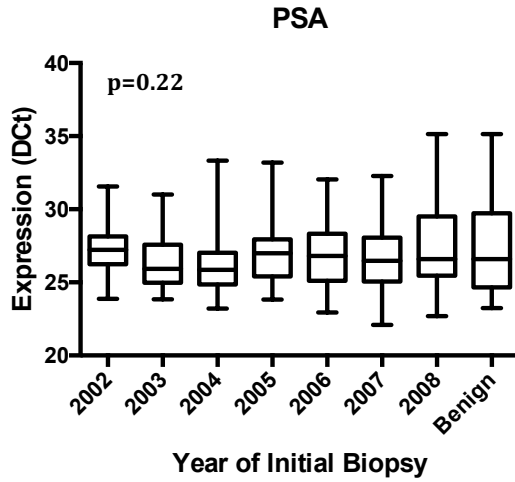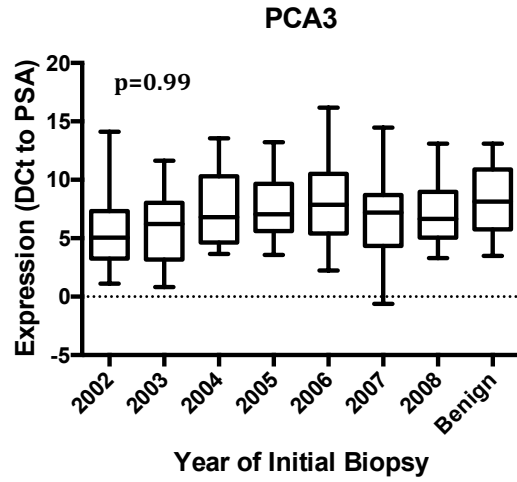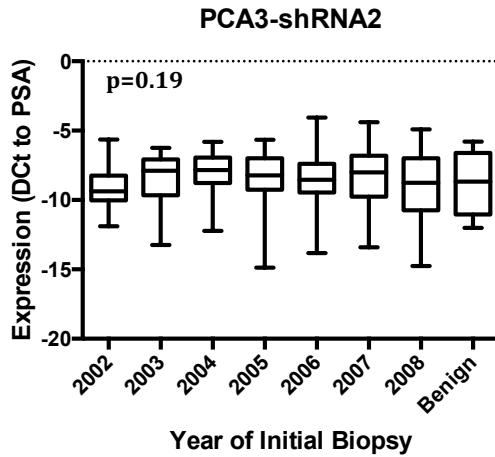

Supplement: S1 Fig — (PDF) [file pone.0175070.s001.pdf]

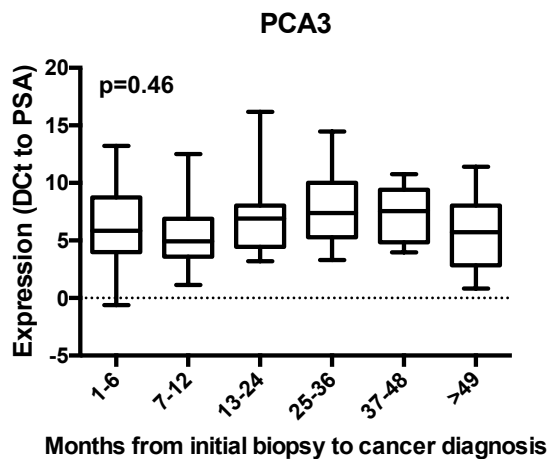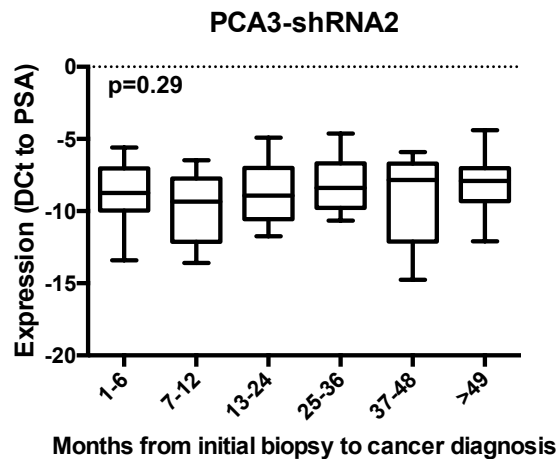

Supplement: S2 Fig — (PDF) [file pone.0175070.s002.pdf]

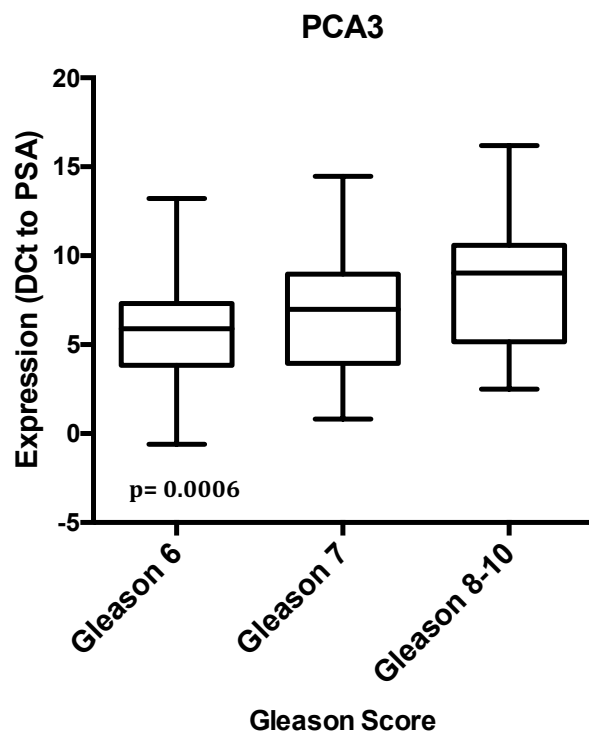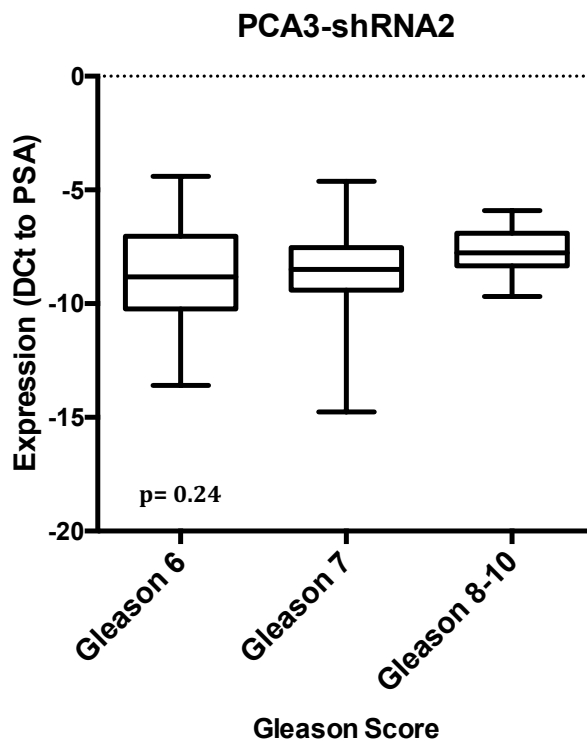

Supplement: S3 Fig — (PDF) [file pone.0175070.s003.pdf]
